# Network Analysis of a Comprehensive Knowledge Repository Reveals a Dual Role for Ceramide in Alzheimer’s Disease

**DOI:** 10.1371/journal.pone.0148431

**Published:** 2016-02-05

**Authors:** Satoshi Mizuno, Soichi Ogishima, Kazuyuki Kitatani, Masataka Kikuchi, Hiroshi Tanaka, Nobuo Yaegashi, Jun Nakaya

**Affiliations:** 1 Department of Clinical Informatics, Tohoku University Graduate School of Medicine, Sendai, Miyagi, Japan; 2 Department of Clinical Record Informatics, Tohoku Medical Megabank Organization, Tohoku University, Sendai, Miyagi, Japan; 3 Department of Gynecology and Obstetrics, Tohoku University Graduate School of Medicine, Sendai, Miyagi, Japan; 4 Department of Genome Informatics, Graduate School of Medicine, Osaka University, Suita, Osaka, Japan; Hungarian Academy of Sciences, HUNGARY

## Abstract

Alzheimer’s disease (AD) is the most common cause of senile dementia. Many inflammatory factors such as amyloid-β and pro-inflammatory cytokines are known to contribute to the inflammatory response in the AD brain. Sphingolipids are widely known to have roles in the pathogenesis of inflammatory diseases, where the precise roles for sphingolipids in inflammation-associated pathogenesis of AD are not well understood. Here we performed a network analysis to clarify the importance of sphingolipids and to model relationships among inflammatory factors and sphingolipids in AD. In this study, we have updated sphingolipid signaling and metabolic cascades in a map of AD signaling networks that we named “AlzPathway,” a comprehensive knowledge repository of signaling pathways in AD. Our network analysis of the updated AlzPathway indicates that the pathways related to ceramide are one of the primary pathways and that ceramide is one of the important players in the pathogenesis of AD. The results of our analysis suggest the following two prospects about inflammation in AD: (1) ceramide could play important roles in both inflammatory and anti-inflammatory pathways of AD, and (2) several factors such as Sphingomyelinase and Siglec-11 may be associated with ceramide related inflammation and anti-inflammation pathways in AD. In this study, network analysis of comprehensive knowledge repository reveals a dual role for ceramide in AD. This result provides a clue to clarify sphingolipids related inflammatory and anti-inflammatory pathways in AD.

## Background

Alzheimer’s disease is the most common cause of senile dementia. Nearly 36 million people were affected by dementia worldwide as of 2010, and this figure is estimated to increase to 65.7 million by 2030 [1]. The societal costs of dementia are already huge and could continue to increase rapidly. Alzheimer’s disease (AD) thus represents a major public health concern and has been identified as a research priority. To address this global social issue, clarification of the pathology and the identification of effective therapies for AD are urgently needed.

The principal pathological feature of AD is brain atrophy and neural cell death. Previous studies demonstrated the occurrence of inflammation in pathologically vulnerable regions of AD brain [2]. Inflammation contributes to pathogenic processes in the degenerating brain tissue [3]. Currently, many inflammatory factors such as amyloid-β and inflammatory cytokines are known to contribute to the inflammatory response in the AD brain [2, 4, 5], and among the inflammatory factors, sphingolipids in particular have been implicated in AD. For instance, altered distributions of the gangliosides GM1 and GM2 [6], elevated levels of ceramide [7] and the up regulation of ceramide generating enzyme [7] have been reported in the AD brain. In addition, sphingolipids such as ceramide and gangliosides are widely known to have roles in the pathogenesis of inflammatory diseases. In cystic fibrosis, an accumulation of ceramide in lung epithelial cells was reported to mediate inflammation and cell death [8]. Ceramide metabolic pathways play vital roles in the pathogenesis of other inflammatory diseases as well, such as inflammatory bowel disease and rheumatoid arthritis [9]. However, the precise roles for sphingolipids in inflammation and neurodegeneration are not well understood in the pathogenesis of Alzheimer’s disease [10].

A comprehensive knowledge repository was proposed to consider global connections among disease factors such as the relationships among signaling molecules, exposures, phenotypes and well-known but not well-understood factors including sphingolipids [11–15]. Such a knowledge repository can provide a comprehensive map of pathogenic signaling pathways based on the ever-increasing data that have accumulated in specific fields such as AD. Each comprehensive knowledge repository includes various types of factors for a disease such as genomic variants, signaling molecules, exposures and phenotypes.

We have previously constructed (AlzPathway 1) and updated a comprehensive map of AD signaling pathways (AlzPathway 2) [16]. It is the first comprehensive knowledge repository, and it was constructed with manual curation from 123 review articles. AlzPathway has been used to simplify the signal transduction pathways of multiple risk factors [17], collect associated factors and pathways of biological interest [18], extract enriched modules among several datasets [19], and provide a training data set in a supervised text mining tool, which warrants future investigation [20]. AlzPathway would also be informative for pathway-based drug discovery efforts [21].

In the present study, 18 review articles related to both sphingolipids and AD were collected and manually curated to update AlzPathway as ‘AlzPathway 3,’ using Cell Designer [22], a modeling editor for biochemical pathways. We also performed a network analysis on AlzPathway 3 to clarify the importance of sphingolipids in AlzPathway and to model relationships among inflammatory factors and sphingolipids in Alzheimer’s disease.

## Materials and Methods

### Update of AlzPathway

We collected all review articles based on these three criteria, 1) related to both sphingolipids and Alzheimer’s disease 2) published after 2000 3) be accessible from PubMed (http://www.ncbi.nlm.nih.gov/pubmed). We obtained 18 review articles [10, 23–39] fulfilling these criteria.

We manually curated these review articles, and then we updated AlzPathway by using Cell Designer. In the process of manual curation, we collect all molecular names and reactions from figures of review articles. We named the updated AlzPathway ‘AlzPathway 3.’ Molecules are distinguished by the following types: proteins, complexes, simple molecules, genes, RNAs, ions, degraded products, and phenotypes. The detailed protocol of the construct relations, file format and gene symbols conformed to the construction method used for AlzPathway [16].

### Construction of binary-relation AlzPathway 3 and extraction of key molecules

We converted AlzPathway 3 from the Systems Biology Graphical Notation (SBGN) process description notation to binary-relation notation. In the SBGN process description notation, a reaction consists of reactants, modifiers, and products. We converted this notation to the binary-relation notation by decomposing reactions into the reactions between (1) reactants and products and those between (2) modifiers and products. For simplification, the molecules were limited to proteins, complexes, genes, RNAs, simple molecules and phenotypes. We calculated the betweenness centralities on the binary-relation AlzPathway 3 to evaluate the topological properties of nodes and edges.

In previous studies, the betweenness centrality [40] was shown to have important meaning in biological networks such as a protein-protein interaction networks [41] and gene regulatory networks [42]. Betweenness centrality is an indicator of how many shortest paths among all nodes in network pass through a node or edge. In AlzPathway, nodes correspond to molecules, and edges correspond to path between two molecules. It was also shown that in AlzPathway, primary factors of AD studies such as amyloid-β and Tau protein have high betweenness centrality [16]. In the present study, we calculated the edge betweenness centrality [43, 44] to extract the high edge betweenness centrality network (primary pathway). We calculated the node betweenness centrality [41] of the binary-relation AlzPathway 3 to extract high centrality nodes and compare the node betweenness centrality between primary factors of AD.

### Evaluation of relationships among sphingolipids and inflammatory factors

A sub-network, namely two hops from “Ceramide” and “Inflammation” was extracted from the binary-relation AlzPathway 3 to model the comprehensive relationships among them. The reason for extracting the sub-network between “Ceramide” and “Inflammation” is that ceramide has high node betweenness centrality next to amyloid-β in AlzPathway 3. Two (hops) is the minimum number to make a connection between “Ceramide” and “Inflammation.” Top 50 high betweenness centrality relations were future extracted from this sub-network to simplify and evaluate the relationships between “Ceramide” and “Inflammation.”

## Results and Discussion

### Overview of AlzPathway 3

**Fig 1:**
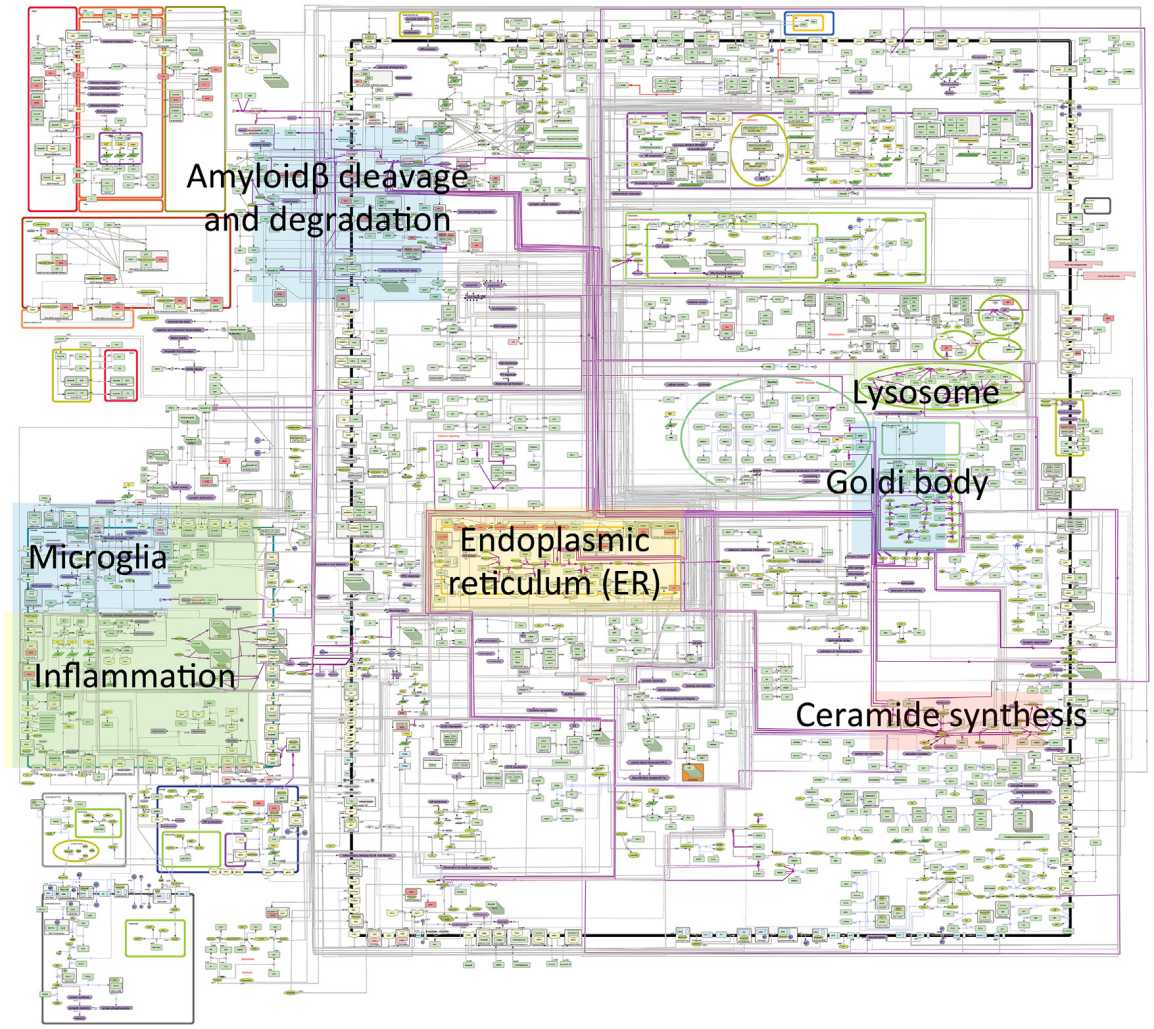
Overview of AlzPathway 3 overlaid with sphingolipid-related canonical pathway annotations. AlzPathway 3 consists of 1,384 molecules, 1,127 reactions, and 138 phenotypes. Purple lines are newly added relations involving sphingolipids.

Here, we present an updated map of AD signaling networks we established (Fig 1). We collected 18 review articles related to both sphingolipids and AD, manually curated these review articles, and updated AlzPathway using Cell Designer ver. 4.2. The AlzPathway 3 map consists of 1,538 species, 1,127 reactions and 138 phenotypes. The molecules shown on AlzPathway 3 can be categorized as follows: 721 proteins, 246 complexes, 300 simple molecules, 33 genes, 37 RNAs, 24 ions, and 23 degraded products. The breakdown of reactions is as follows: 472 state transitions, 22 transcriptions, 30 translations, 184 heterodimer associations, 56 dissociations, 106 transports, 22 unknown transitions, six unknown negative influences and 226 omitted transitions. In AlzPathway 3, sphingolipid-involved relations can be found in the following canonical pathways: “amyloid-β cleavage and degradation”, “inflammation”, “ganglioside synthesis in the endoplasmic reticulum”, “sphingolipids metabolism in lysosomes”, and “sphingolipid synthesis in Goldi network” and “ceramide synthesis” (Fig 1). The AlzPathway 3 is available as the SBML map for CellDesiginer (S2 File). The latest version of AlzPathway map is accessible at http://www.alzpathway.org/.

### Binary-relation notation and key molecules

**Fig 2:**
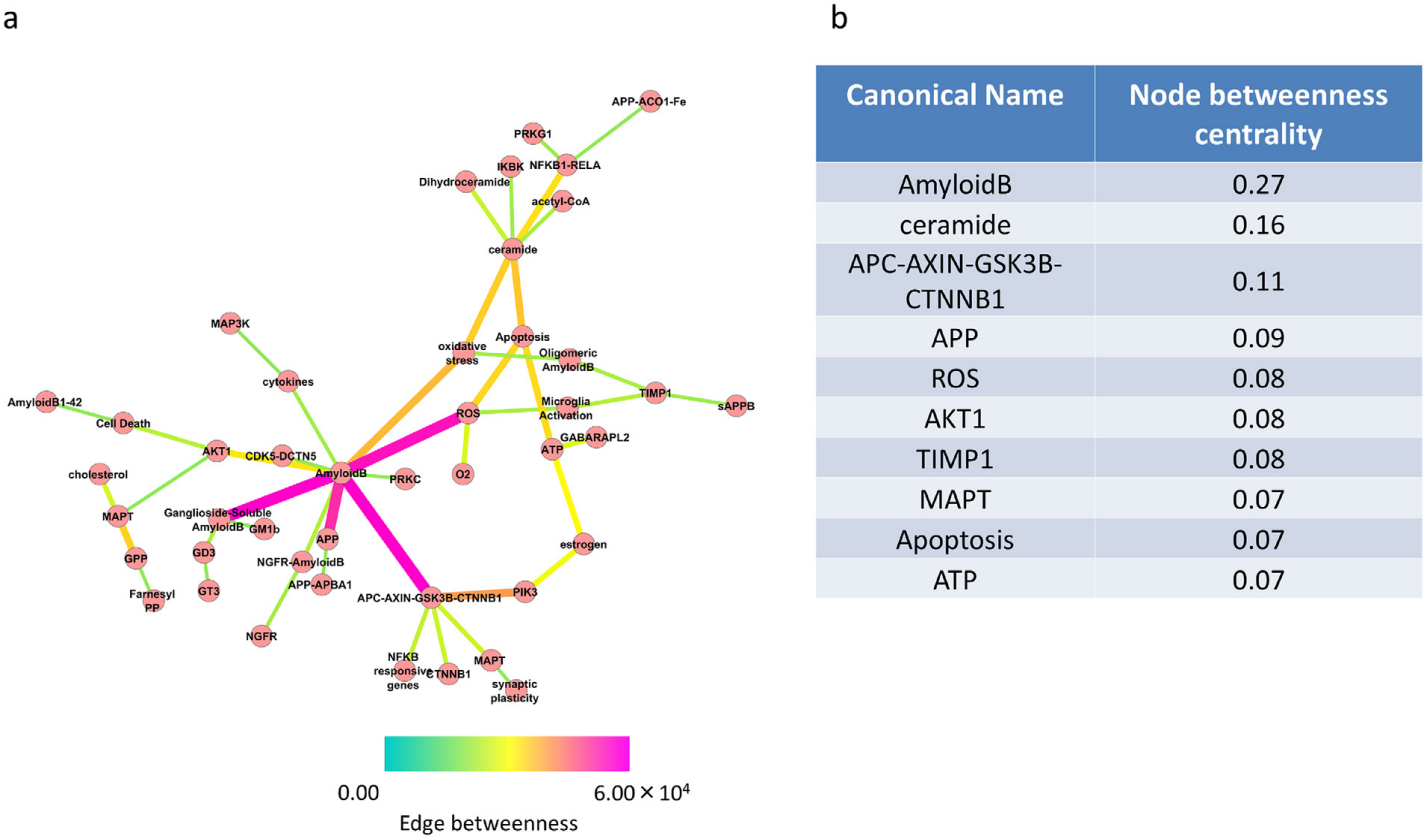
Binary-relation notation AlzPathway 3 and the key molecules. (a) The top 50 high-centrality relations as the highlighted primary pathway of AlzPathway. Circles are nodes in AlzPathway 3. Lines are edges between nodes. As represented, red lines have high edge betweeness centrality and blue lines have low. (b) The top 10 high-betweenness centrality nodes and their centrality.

To clarify the principal structure of AlzPathway 3, we constructed the binary-relation notation AlzPathway 3. Binary-relation notation AlzPathway 3 is available as the cys file (S3 File) which can be opened by Cytoscape 3. [45] High-centrality relations were highlighted as primary relations in accordance with the edge betweenness centrality of each reaction. The top 50 high-centrality relations are shown in Fig 2a. Among the several highlighted binary relations were AD hallmark pathways that are the same as in AlzPathway [16]: amyloid plagues (amyloid-β accumulation), i.e., relations involving amyloid-β, and neurofibrillary tangle accumulation (hyper-phosphorylated tau accumulation), i.e., relations connecting amyloid-β precursor protein (APP), APC-AXIN-GSK3β-CTNNB1 complex and microtubule-associated protein tau. Some less known relations were also found among the highlighted binary relations: those connecting ceramide and amyloid-β. In the ranking of node betweenness (Fig 2b), amyloid-β is the highest centrality node (0.27) and ceramide is the second- highest (0.16).

These results for the centralities of binary-relation notation AlzPathway 3 show that pathways related to ceramide are some of the primary pathways, as are those involving amyloid plagues and neurofibrillary tangle accumulation. In addition, the results of our network analysis suggest that ceramide is one of the important players in the pathogenesis of AD, as shown in previous studies [46–48].

### Sphingolipids and inflammation mediators in AlzPathway

**Fig 3:**
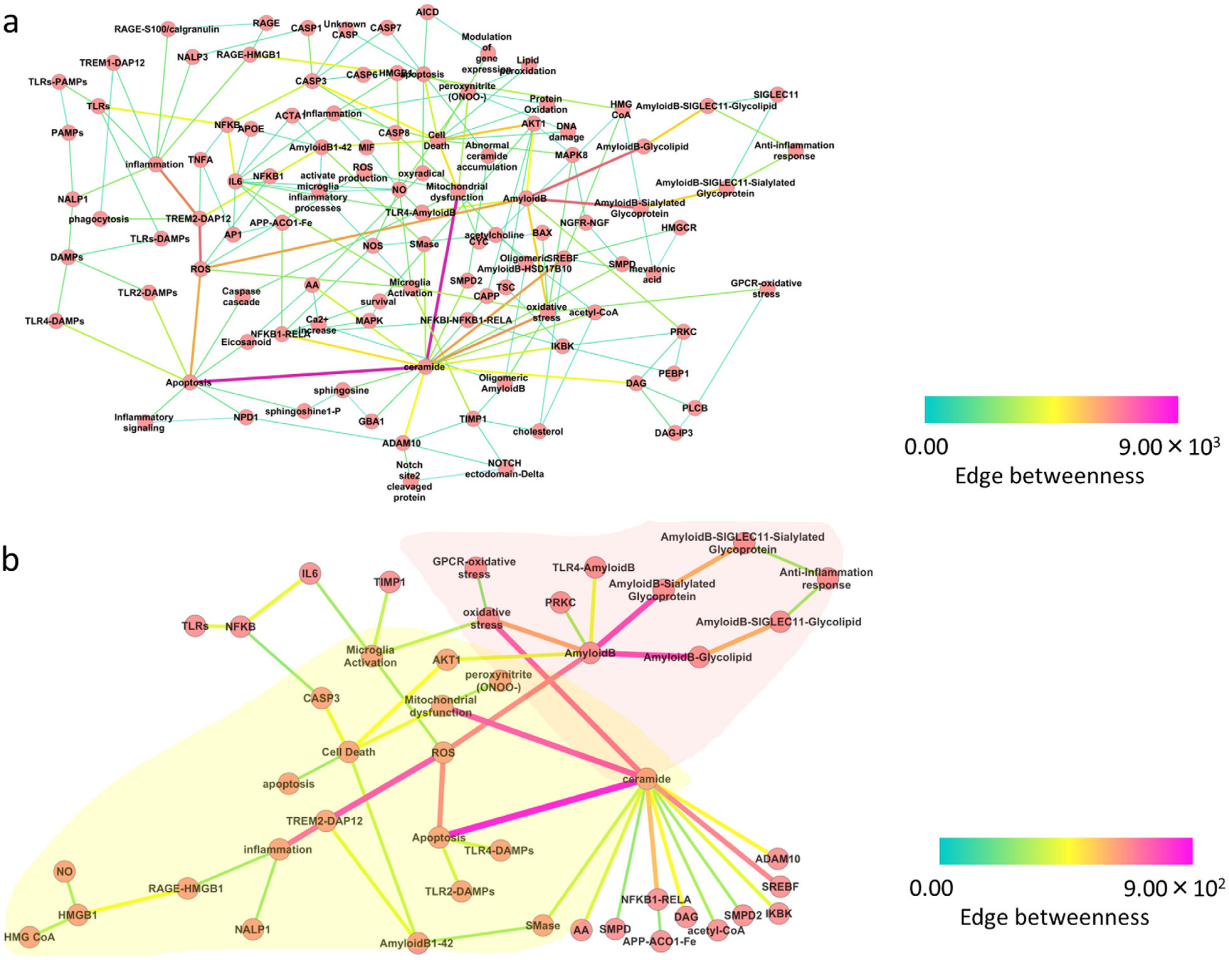
Factors potentially associated with ceramide and inflammation. (a) Extracted sub-network from the binary-relation notation AlzPathway 3. The simplified binary-relation notation AlzPathway 3 consists of two hops from “Ceramide” and “Inflammation.” (b) The top 50 high-centrality relations of the simplified binary-relation notation AlzPathway 3.

Ceramide is one of the highest centrality nodes in binary-relation notation AlzPathway 3. We extracted a sub-network consisting of two hops from “Ceramide” and “Inflammation” from binary-relation notation AlzPathway 3 to model the comprehensive relationships among ceramide and inflammatory mediators (Fig 3a). The sub-network termed as ‘simplified binary-relation notation AlzPathway 3.’ Based on the edge betweenness centrality of each reaction, the high-centrality relations were highlighted as primary relations. The top 50 high-centrality relations in simplified binary-relation notation AlzPathway 3 are shown in Fig 3b.

We found two inflammation-related pathways in simplified binary-relation notation AlzPathway 3: (1) an inflammatory pathway connecting ceramide and “Inflammation” (overlaid by yellow), and (2) an anti-inflammatory pathway connecting ceramide and “Anti-inflammation response” (overlaid by red). The inflammatory pathway includes several proteins and phenotypes: TREM-DAP12 complex, sphingomyelinase, amyloid-β1–42, mitochondria dysfunction, microglia activation and apoptosis. TREM-DAP12 complex is the starting point in the inflammatory pathway, which is a well-known trigger of inflammation [49]. Previous studies showed that the secretion of sphingomyelinase is triggered by inflammatory cytokines [50] and that the expression of sphingomyelinase is upregulated in tissues/cells of inflammatory diseases including AD [7, 51]. Amyloid-β1–42 is known as a stimulator of neuroinflammation in the AD brain [52]. These studies suggest that TREM-DAP12 complex, sphingomyelinase and amyloid-β1–42 play roles in inflammatory signaling in the AD brain.

In contrast, the anti-inflammatory pathway includes several nodes showed as amyloid-β-, glycolipid- and glycoprotein-involved relations. In these relations, amyloid-β-glycolipid, amyloid-β-sialylated glycoprotein, and amyloid-β- sialic acid-binding immunoglobulin-type lectin (iglec) 11-glycolipid and amyloid-β-siglec 11-sialylated glycoprotein complexes were found. These siglec 11 complexes lie on the membrane of microglia and reduce the induction of interleukin (IL)-1β and inducible nitric oxide synthase [53]. This reduction activity leads to a suppression of microglial inflammation.

These studies revels that ceramide has both proinflammatory and anti-inflammatory effects in microglia. Our network analysis suggests two possibilities regarding inflammation in AD: (1) ceramide is centeredin both inflammatory and anti-inflammatory pathways of AD, and (2) several factors such as sphingomyelinase and siglec 11 are associated with inflammation or anti-inflammation, respectively.

The regulatory mechanisms of inflammation and anti-inflammation by ceramide and inflammatory mediators will be revealed by further studies.

In conclusion, we showed the importance of ceramide in the pathogenesis of AD. We have collected and manually curated review articles related in AD, and we updated AlzPathway using Cell Designer named it AlzPathway 3. Our network analysis revealed that ceramide in particular among the sphingolipids is a key molecule in AD. Ceramide could play important roles in both inflammatory and anti-inflammatory pathways. The results of our network analysis also suggest mediators of inflammation and anti-inflammation in addition to ceramide in AD. These results contribute to a new hypothesis that ceramide play a dual role in the regulation of inflammation in AD brain and need further investigation as a mechanism of inflammatory mediation in the AD brain. The result of network analysis provides a clue to clarify inflammatory and anti-inflammatory pathway related sphingolipids in AD. Our comprehensive knowledge repository and network analysis approach will be used to increase the database for AD drug discovery and development.

### Comparing with other knowledge driven strategies

Recently, a study with network analysis of knowledge driven protein-protein interaction (PPI) network has been reported [54]. In this study, they calculated reliability scores of PPI with knowledge and discovered ‘Knowledge cliff’ which includes new therapeutic target of AD. Their research was specialized to discover therapeutic target with PPI rather than clarify AD related molecular mechanisms. Our AlzPathway focuses on not only PPI but also on signaling cascade including small molecules such as sphingolipids. Because of this completeness, AlzPathway is providing clues to clarify comprehensive molecular mechanisms such as network analysis on this research.

## Supporting Information

S1 File SBML map file of AlzPathway3. The SBML map file alzpathway_3 can be browsed using CellDesigner. Please download CellDesigner at http://www.celldesigner.org/, install it, and open the SBML map file alzpathway_sbml_map.xml to browse AlzPathway map by CellDesigner.
(XML)

S2 File Binary-relation notation file of AlzPathway 3. The cys file alzpathway_br.cys is the binary-relation notation of AlzPathway 3 which can be opened by using Cytsoscape 3.
(ZIP)

S1 Table Gene names and their functional categories which have been newly added to the AlzPathway 3. (XLSX)
